# Bacteriology and Surgical Technic for Nurses

**Published:** 1911-01

**Authors:** 


**Bacteriology and Surgical Technic for Nurses.** By **Emily M. A. Stoney,** Superintendent of Training School for Nurses, Rock Island, Ill. Third edition. 12 mo, pp. 311. Illustrated. Philadelphia and London. W. B. Saunders Co. 1910. (Cloth, $1.50.)

This is a companion volume of "Practical Points in Nursing" by the same author. The third edition has been revised and enlarged by Frederick Richardson Griffith, M.D. The first part of the book is devoted to bacteriology and antiseptics, the second part to surgical technic. Signs of death, and autopsies are considered in one chapter. Some well tried receipts on diet have been added, and special surgical operations have been considered a little more fully in this revision. The chapter on preparation of the patient for operation, and care of the patient during and after operation is most excellent and well worth reading. J. A. R.

